# Predict: surveillance and prediction for emerging pathogens of wildlife

**Published:** 2011-01-10

**Authors:** William B Karesh

**Affiliations:** 1 EcoHealth Alliance, 460 West 34th St., 17th Floor, New York, NY. 10001, USA; 2 OIE Working Group on Wildlife Diseases; 3 IUCN Species Survival Commission - Wildlife Health Specialist Group; 4 Emerging Pandemic Threats - PREDICT program

## 

Nowhere in the world are the health impacts from emerging diseases of wildlife more important than in developing countries, where daily work and livelihoods are highly dependent on natural resources. Many of these same countries have little to no capacity for detecting disease emergence in wildlife and domestic animals prior to spread to humans. While the linkages of human, animal, and environmental health is at the heart of the One Health approach, an increasingly important prism through which governments, NGOs, and practitioners view public health, we still have three critically important challenges facing us: 1) we need a much broader and deeper knowledge of what pathogens are waiting to emerge from the animal kingdom, 2) we need to better target our investigations to maximize available resources, and 3) we need better tools to predict or determine if an organism is a pathogen of significance for causing human disease. The PREDICT project of the USAID Emerging Pandemic Threats program is endeavoring to build capacity for surveillance of emerging diseases in wildlife in order to help to address these three challenges.

